# Locking-to-unlocking system is an efficient strategy to design DNA/silver nanoclusters (AgNCs) probe for human miRNAs

**DOI:** 10.1093/nar/gkv1377

**Published:** 2015-12-17

**Authors:** Pratik Shah, Suk Won Choi, Ho-jin Kim, Seok Keun Cho, Yong-Joo Bhang, Moon Young Ryu, Peter Waaben Thulstrup, Morten Jannik Bjerrum, Seong Wook Yang

**Affiliations:** 1 UNIK Center for Synthetic Biology, University of Copenhagen, Thorvaldsensvej 40, DK-1871 Frederiksberg C, Copenhagen, Denmark; 2 Seoulin Bioscience Co. Ltd. 4F. #A, KOREA BIO PARK, 700, Daewangpangyo-ro, Bundang-gu, Seongnam-si, Gyeonggi-do, Korea; 3 Department of Chemistry, University of Copenhagen, Universitetsparken 5, DK-2100, Copenhagen, Denmark; 4 Department of Systems Biology, College of Life Science and Biotechnology, Yonsei University, Korea

## Abstract

MicroRNAs (miRNAs), small non-coding RNA molecules, are important biomarkers for research and medical purposes. Here, we describe the development of a fast and simple method using highly fluorescent oligonucleotide-silver nanocluster probes (DNA/AgNCs) to efficiently detect specific miRNAs. Due to the great sequence diversity of miRNAs in humans and other organisms, a uniform strategy for miRNA detection is attractive. The concept presented is an oligonucleotide-based locking-to-unlocking system that can be endowed with miRNA complementarity while maintaining the same secondary structure. The locking-to-unlocking system is based on fold-back anchored DNA templates that consist of a cytosine-rich loop for AgNCs stabilization, an miRNA recognition site and an overlap region for hairpin stabilization. When an miRNA is recognized, fluorescence in the visible region is specifically extinguished in a concentration-dependent manner. Here, the exact composition of the fold-back anchor for the locking-to-unlocking system has been systematically optimized, balancing propensity for loop-structure formation, encapsulation of emissive AgNCs and target sensitivity. It is demonstrated that the applied strategy successfully can detect a number of cancer related miRNAs in RNA extracts from human cancer cell lines.

## INTRODUCTION

In eukaryotes, microRNAs (miRNAs) are crucial key regulators in multiple cellular processes, because of their fundamental roles in gene expression achieved through target mRNA clearance or suppression of the translation processes (1–4). Expression of almost all miRNAs is temporally and spatially regulated in specific tissues, during specific stages of cell development, cell growth, or in response to diseases such as cancers. The number of known miRNAs is quite large; there are an estimated 2500 in humans and even in a short-lived model plant such as *Arabidopsis* more than 400 miRNAs are known. In order to monitor the expression levels of miRNAs, a variety of methods have been developed during the last two decades, including widely used conventional methods and advanced technology based methods (5–11). To address the challenge of quick and specific recognition of miRNA, we have developed a method exploiting the photochemical characteristics of DNA-bound silver nanoclusters, herein named DNA/AgNCs-based miRNA detection method. By exploiting short DNA oligonucleotides as a template, tunable, stable and strong photoluminescence properties of AgNCs have been achieved for applications in biological systems. Many studies have reported that DNA encapsulated silver nanoclusters (DNA/AgNCs) probes are competent to detect target nucleic acids (12–14), proteins (15,16), single nucleotide polymorphisms (17–19) and chemical analytes including ATP (20) and drugs (21). Similarly, we recently showed that a series of designed DNA/AgNCs probes can be applied to detect miRNAs (22). The DNA/AgNCs probes consist of a DNA scaffold and a DNA sequence complementary to target miRNA. In the presence of target miRNA, the strong fluorescence of DNA/AgNCs is switched off. Quantitative detection of target miRNA and can be monitored within an hour of reaction (22). Fluorescent oligonucleotide-stabilized silver nanoclusters in general are not well characterized yet with regard to their exact nature in terms of structure, charge and binding mode, although there are many interesting results and models published (23–26). From experience, a non-emissive DNA/AgNCs probe (lacking a well-defined structure) can be converted into a highly emissive DNA/AgNCs probe by sequence adjustment to allow for hairpin or self-dimer formation (27,28). Previously, it was suggested that sequence trimming of particular 12-mer DNA nucleotides could yield a ‘color pallet’ of so-called DNA-12nt scaffolds with varied emission properties (29). Yet, DNA/AgNCs probes with such DNA-12nt scaffolds extended by a target recognition sequence, mostly gave emission confined between the red and near infrared spectral range. As a rare case, we observed that a combining of a DNA-12nt scaffold with a target complementary sequence for miR396 generated a strong green emission (28). These results imply that the whole sequence profile of a given probe is important for the optical properties of the AgNCs. On this background we made incremental improvements on DNA/AgNCs probes based on DNA-12nt scaffolds, but found that adaption to a specific sequence of miRNA often required a high frequency of trial and errors because of unpredictable structure formation, *vide infra*.

Here, we suggest a new, robust design strategy, a locking-to-unlocking system, exploiting a fold-back anchored DNA template for emissive AgNCs encapsulation addressing the challenge of quick and specific recognition and quantification of miRNAs. The strong point of this strategy consists in its structural uniformity, by which the secondary structure of DNA templates can be confined, and that enables us to more conveniently design DNA/AgNCs probes. Indeed, using this strategy, we readily designed three different fold-back anchored DNA/AgNCs probes and further tested their functionality *in vitro*. Moreover, all three tested probes are highly competent to detect several target miRNAs such as miR-18a, miR-21 and miR-27b in total RNA samples extracted from human cancer cell lines.

## MATERIALS AND METHOD

### Synthesis of target miRNAs

DNA probes and desalted miRNA targets were obtained from IDT (Integrated DNA Technologies, BVBA. Interleuvenlaan 12A, 3001 Leuven, Belgium). The synthesis of emissive AgNCs was carried out using AgNO_3_ (> 99.99%) and NaBH_4_ (99.99%) from Sigma–Aldrich. A sodium nitrate solution and a Tris-Acetate buffer (pH 7, 0.5 M) from TRIZMA® acetate salt (≥99.0%, from Sigma–Aldrich) were prepared in pure MilliQ water (18.2 MΩ cm).

### AgNCs synthesis procedure, sample preparation and fluorescence detection

**Figure 1.**
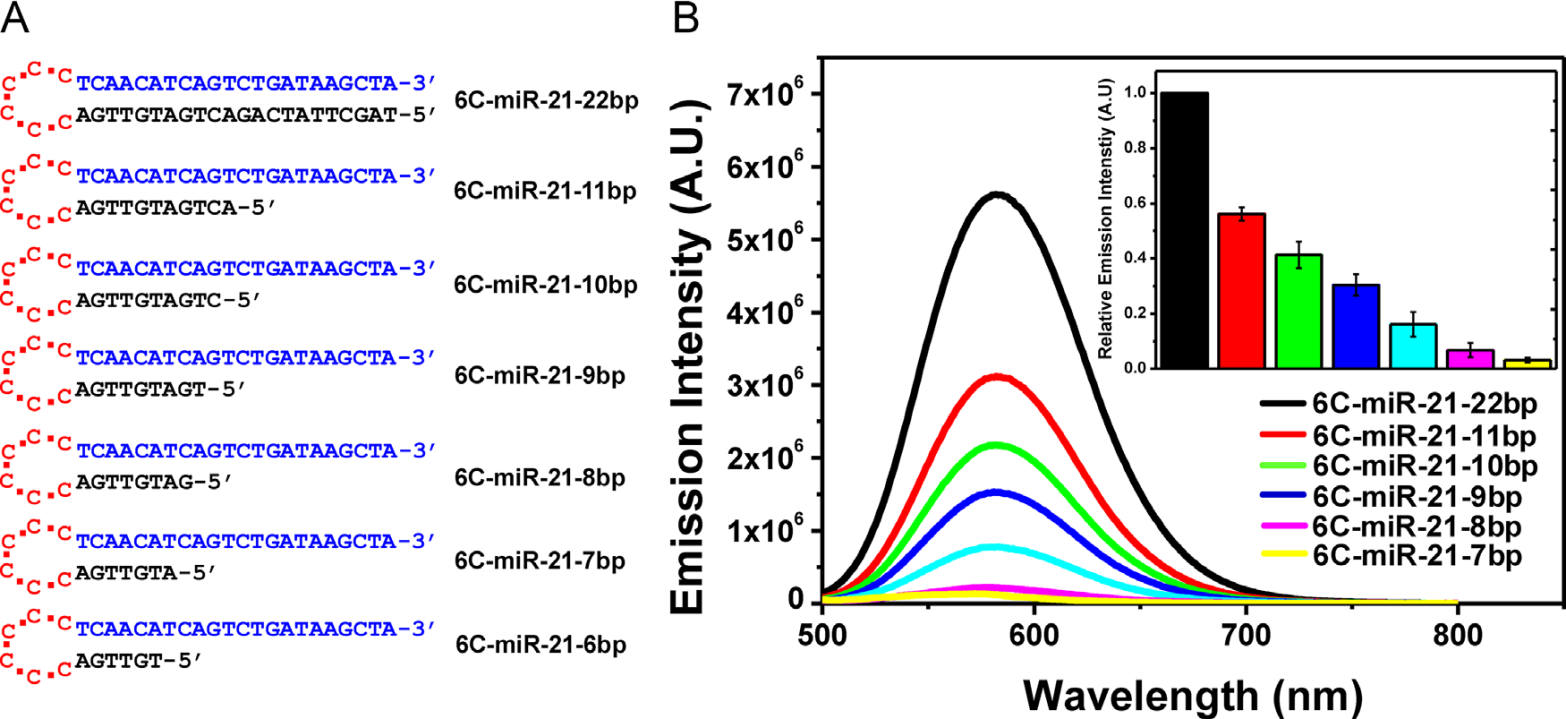
(**A**) Sequences of the seven DNA probes with varied length of the anchor sequence. Each DNA probe sequence is given in three colors. Red sequences: cytosine-loop (6C), blue sequences: target miRNA sensing sequence (miR-21) and black sequences: the anchor sequence complementary to target sensing sequence. (**B**) Emission spectra of the seven DNA/AgNCs probes. Inset shows the relative emission intensity in response to the reduced length of the anchor sequence. The data were collected as average value of three individual experiments with ±standard deviation (*n* = 5).

The DNA and miRNA sequences used in the publication are described in Figures 1A, 6A, and Supplementary Figure SI 1,2,3,8 and Supplementary Table S1. To make fluorescent AgNCs, we incubate the DNA probes (25 μl) at 95°C for 10 min (denaturation) and at 25°C for 20 min (annealing) in the given concentrations of 20 mM Tris-acetate buffer and 25 mM NaNO_3_. Then, AgNO_3_ and NaBH_4_ were added to the final concentration of 250 μM in 50 μl reaction volume. In addition to the DNA/AgNCs of interest, the synthesis procedure inevitably leads to formation og silver nanoparticles with absorbance in the 400 nm range as well as minor fluorescent species. During sample preparation all operations should be performed in a systematic manner obtain the optimal results. Throughout we designate the concentrations of nucleic acids, buffer and salts in the final reaction volume (50 μl). All the DNA/AgNCs were incubated for 1 h at 25°C and subsequently used for further analysis. For high pressure liquid chromatography (HPLC) separation experiments (see below), the synthesis was scaled up 4-fold to yield a final reaction volume of 200 μl. For fluorescence spectroscopy, the 50 μl samples were diluted with 450 μl of distilled water before measurement on a Horiba Jobin Yvon, Fluoromax-4 fluorimeter in a 10 mm disposable cuvette (5 nm slit for excitation and 10 nm for emission). For absorbance and circular dichroism spectroscopy (see below), the dilution was only by a factor 4 to yield a final volume of 200 μl. For fluorescence spectroscopy of animal samples, the 50 μl samples were diluted with 100 μl of distilled water before measurement on a CLARIOstar microplate reader (BMG LABTECH, Ortenberg, Germany). All spectroscopic measurements were performed at room temperature (25°C).

**Figure 2.**
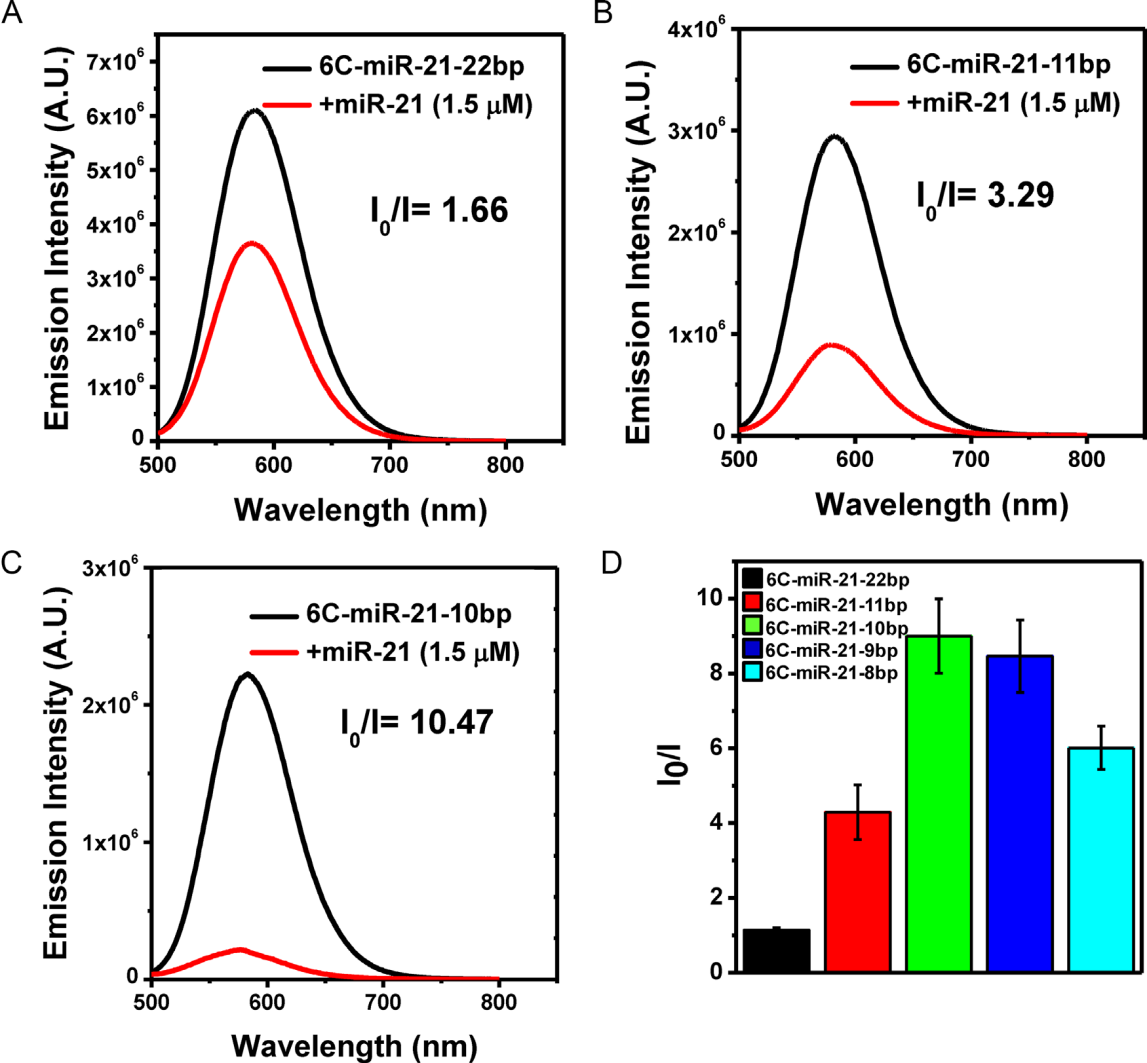
(**A**) Target sensitivity of the DNA probe 6C-miR-21–22bp (1.5 μM) in the absence (black curve) and presence of target miR-21 (1.5 μM) (red curve). (**B**) Target sensitivity of the DNA probe 6C-miR-21–11bp (1.5 μM) in the absence (black curve) and presence of target miR-21 (1.5 μM) (red curve). (**C**) Target sensitivity of the DNA probe 6C-miR-21–10bp (1.5 μM) in the absence (black curve) and presence of target miR-21 (1.5 μM) (red curve). (**D**) Target sensitivity assessment of the DNA probes of varied anchor length measured as the emission spectra obtained following excitation and emission at 480 and 580 nm, respectively. 1.5 μM of miR-21 was mixed with of 6C-miR-21–22bp (1.5 μM) (black bar), 6C-miR-21–11bp (1.5 μM) (red bar), 6C-miR-21–10bp (1.5 μM) (green bar), 6C-miR-21–9bp (1.5 μM) (blue bar) or 6C-miR-21–8bp (1.5μM) (sky-blue bar). *I*_0_/*I* refer to the emission intensity of DNA probes in the absence (*I*_0_) and presence (*I*) of target miRNA. The data were collected as average value of three individual experiments with ±standard deviation (*n* = 5).

### Structural implications of fold-back anchored DNA/AgNCs

**Figure 3.**
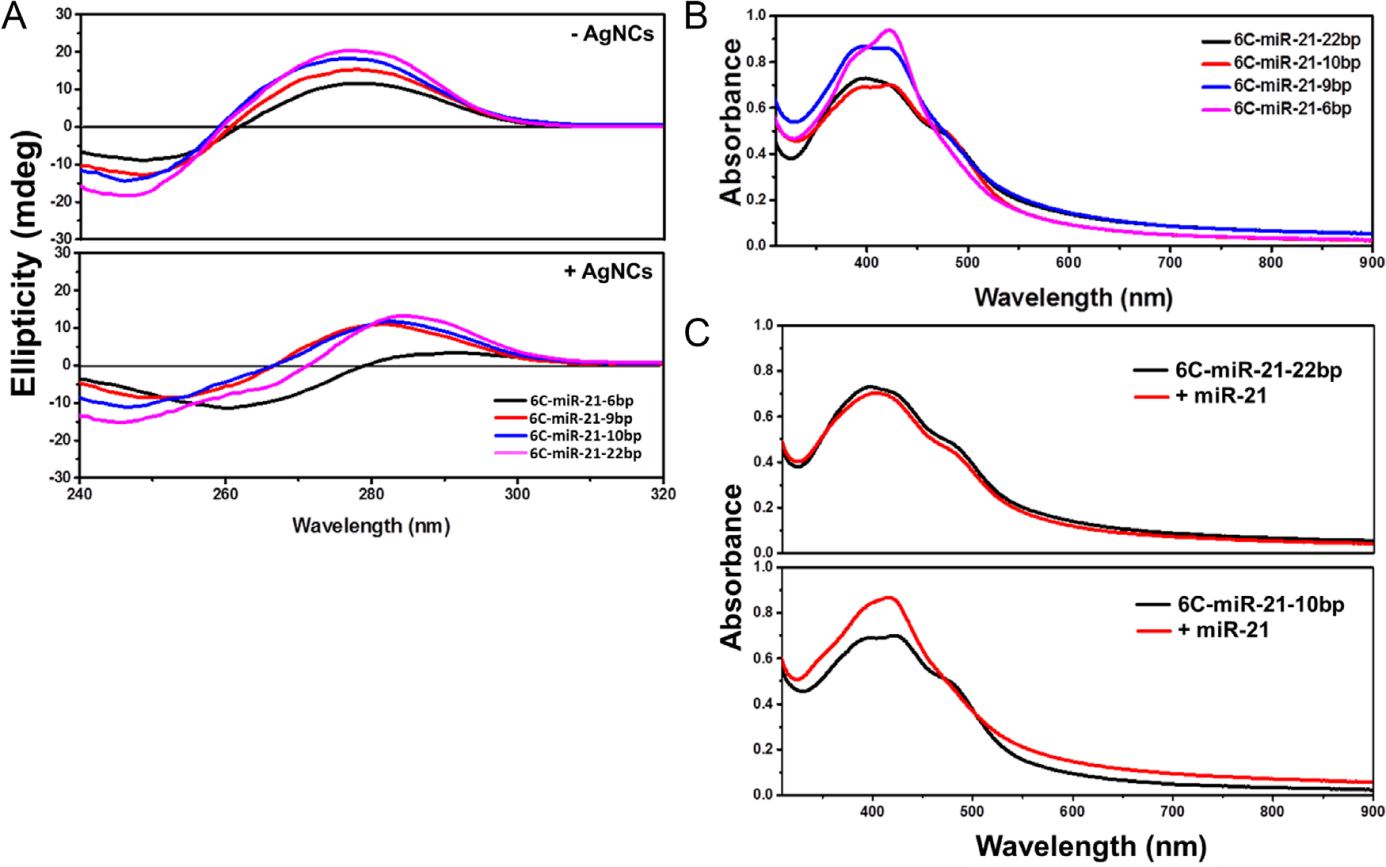
(**A**) UV-circular dichroism (CD) spectra of fold-back anchor DNA/AgNCs probes. Top panel: CD spectra of fold-back anchor DNA/AgNCs probes without AgNCs. Bottom panel: CD spectra of fold-back anchor DNA/AgNCs probes with AgNCs. (**B**) Absorption spectra of fold-back anchor DNA/AgNCs probes with a different anchor in length. (**C**) Absorption spectra of 6C-miR-21–22bp and 6C-miR-21–10bp in the presence or absence of target miR-21. Top panel: absorption spectra of 6C-miR-21–22bp without miR-21 (black curve) or with miR-21 (red curve). Bottom panel: absorption spectra of 6C-miR-21–10bp without miR-21 (black curve) or with miR-21 (red curve). For (B) and (C) please note that the absorbance around 400 nm is attributed to the presence on non-emissive silver nanoparticles.

The annealed samples of the short DNA sequences of the probes, yield species in solution that may be distributed between extended monomers and hairpins. In Figure 3A, the UV circular dichroism spectra of 6C-miR-21–22bp, 6C-miR-21–10bp, 6C-miR-21–9bp and 6C-miR-21–6bp are shown before (top) and after (bottom) the reaction to form AgNCs. It can be observed that all spectra in Figure 3 A on unmodified samples show a similar trend with a positive peak at 280 nm and a zero-crossing around 255 nm, with a negative peak at lower wavelength. The observed CD pattern is in accordance with a B-form type folding (44). It is notable that except for 6C-miR-21–6bp, the CD after formation of AgNCs (Figure 3A, bottom) follows a similar trend, indicating that the nucleic acids in the samples with a 9, 10 and 22 bp overlap have not been extensively refolded by the presence of AgNCs. In Figure 3B, the absorbance in the visible region is shown for the same four probes; it is noteworthy that the shoulder at 480 nm is not observed in 6C-miR-21–6bp, in agreement with the low emission of this probe. In Figure 3C (top), it is shown that addition of miR-21 to 6C-miR-21–22bp does not lead to an altered absorbance spectrum, which can be correlated with the unchanged fluorescence emission. On the other hand as shown in Figure 3C (bottom), the 6C-miR-21–10bp probe loses the 480 nm shoulder in the presence of miR-21, underlining the importance of this feature as a characteristic of an emissive species.

**Figure 4.**
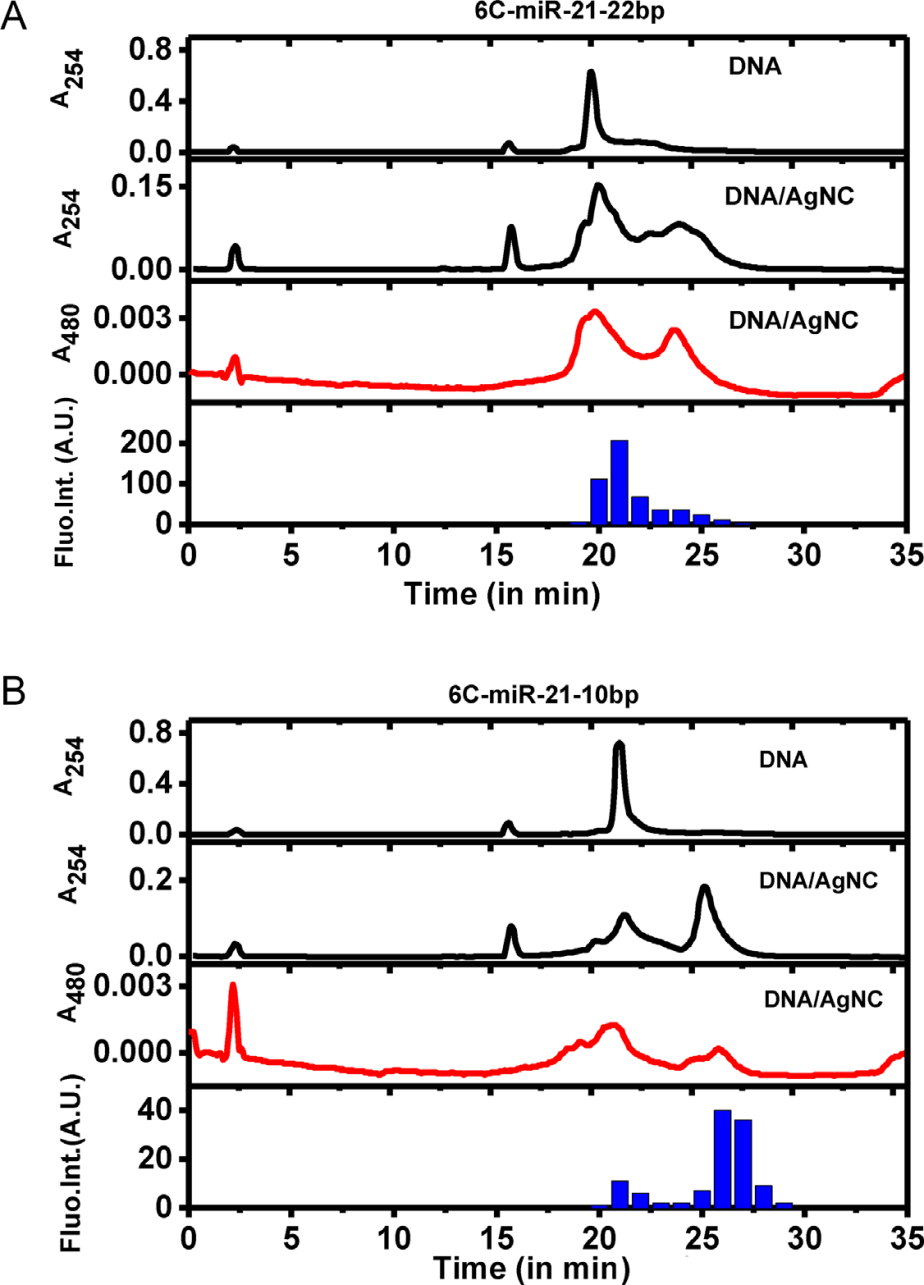
HPLC chromatograms of the separation of unmodified and AgNCs modified 6C-miR-21–22bp and 6C-miR-21–10bp. (**A**) Top: chromatograms of unmodified and AgNCs modified 6C-miR-21–22bp with absorbance measured at 254 nm wavelength (shown in black). Middle: chromatograms of AgNCs modified 6C-miR-21–22bp with absorbance measured at 480 nm wavelength (shown in red). Bottom: blue bar graph showing fluorescence of collected samples of AgNCs modified 6C-miR-21–22bp. (**B**) Top: chromatograms of unmodified and AgNCs modified 6C-miR-21–10bp with absorbance measured at 254 nm wavelength (shown in black). Middle: chromatograms of AgNCs modified 6C-miR-21–10bp with absorbance measured at 480 nm wavelength (shown in red). Bottom: blue bar graph showing fluorescence of collected samples of AgNCs modified 6C-miR-21–10bp.

Further insight was obtained by proceeding with the HPLC separation of unmodified and AgNCs modified 6C-miR-21–22bp and 6C-miR-21–10bp, as shown in Figure 4. It is observed from the separations of unmodified 6C-miR-21–22bp (Figure 4A) and 6C-miR-21–10bp (Figure 4B) that the samples contain two or more different conformations of the DNA at varying concentrations. This indicates that 6C-miR-21–10bp and 6C-miR-21–22bp can adopt more than one conformation in solution and that annealing is an important step in the preparation of AgNCs modified sensors. After modification of the DNA with AgNCs several peaks show up in the chromatograms, grouped around two major peaks. In the case of 6C-miR-21–22bp (Figure 4A), the fluorescence is found in the first peak, while in the case of 6C-miR-21–10bp (Figure 4B) the fluorescence is found in the latter of the two peaks on the chromatogram. This indicates that the fraction of 6C-miR-21–22bp modified with AgNCs that shows fluorescence (Figure 4A) has a structure quite similar to the major fraction of unmodified DNA. Opposite to this we find that the 6C-miR-21–10bp modified with AgNCs that shows fluorescence (Figure 4B) probably has a structure different from the major fraction of unmodified DNA. Another interesting point is that the absorption spectra of the sample, where fluorescence is observed, is very different from the spectra of the sample both before and after in the chromatogram. The peak showing fluorescence has a distinct visible absorption peak around 480 nm with reduced absorption around 350–450 nm while the absorption before and after were in the range 350–450 nm with little absorption around 480 nm (data not shown). This is observed for both 6C-miR-21–10bp and 6C-miR-21–22bp. This could indicate that the fluorescence is connected to a specific AgNCs structure with an absorption peak around 480 nm and that this structure is the same in the two DNA structures. Also, it is a strong indication that the absorbance at 350–450 nm seen in Figure 4B and C does not arise due to the presence of fluorescent DNA/AgNCs, which may suggest that the applied synthetic procedure can be improved. However, despite this, the obtained products are nevertheless capable of a sensitive and selective miRNA detection even in a complex mixture (*vide infra*).

### Optimized fold-back anchored DNA/AgNCs probe for miR-21 shows efficacious target sensitivity and specificity

**Figure 5.**
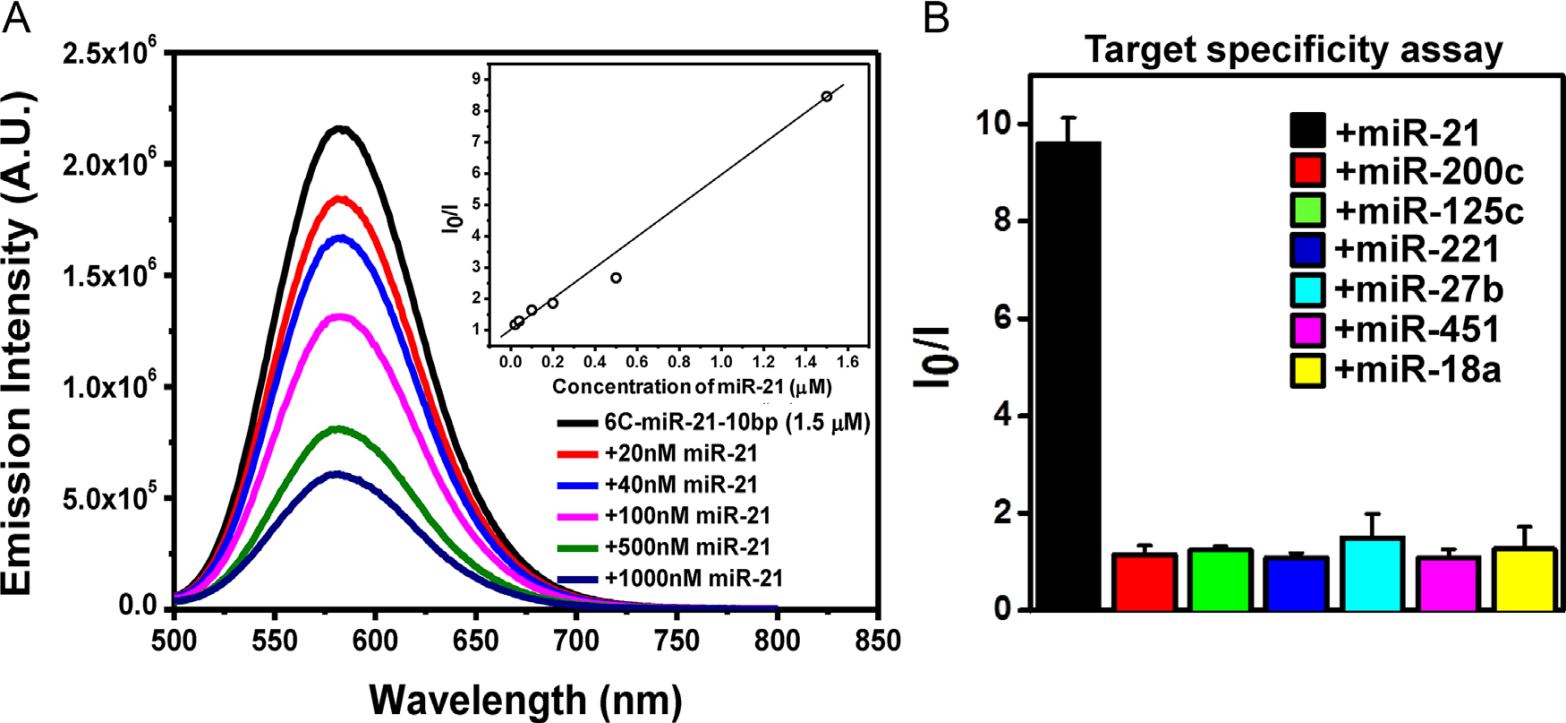
(**A**) Fluorescence intensity of the 6C-miR-21–10bp DNA probe after addition of target miR-21 in a concentration ranging from 0 to 1.0 μM. The fluorescence spectra were recorded, exciting at 480 nm. The inset shows the Stern–Volmer plot. (**B**) Specificity assessment of the 6C-miR-21–10bp DNA probe towards different miRNAs measured as the emission spectra obtained following excitation at 480 nm. 1.5 μM 6C-miR-21–10bp probe was mixed with 1.5 μM of miR-21 (black bar), miR-200c (red bar), miR-125c (green bar), miR-221 (blue bar), miR-27b (sky blue bar), miR-451 (pink bar) and miR-18a (yellow bar). The data were collected as average value of three individual experiments with ±standard deviation (*n* = 5).

As the logical next step, the fold-back anchored DNA/AgNCs probes were tested for the ability to recognize target miRNA in a concentration dependent manner. As shown in Figure 5A, the presence of an increasing concentration of miR-21 led to a decrease in the yellow fluorescence of 6C-miR-21–10bp. The inset shows that the Stern–Volmer plot of the data in Figure 5A follows a linear dependence of the *I*_0_/*I* intensity versus miR-21 target concentration (*I*_0_ being the emission value in the absence of miR-21). This result indicated that 6C-miR-21–10bp is able to detect its target to least 20 nM, corresponding to an amount of 10 picomoles in the 500 μl sample volume (Figure 5A). Further, we tested the target specificity of the DNA/AgNCs probes by adding a mixture of non-specific miRNAs. The 6C-miR-21–10bp probe was incubated with several non-target miRNAs such as miR-200c, miR-125c, miR-221, miR-27b, miR-451 and miR-18a (Figure 5B). Similar to the results in our previous reports (27,32), the newly designed 6C-miR-21–10bp probe successfully distinguished its specific target miR-21 from the non-specific miRNAs. As shown in Figure 5B, the 6C-miR-21–10bp probe displayed an *I*_0_/*I* value of 9 in the presence of its target, but it showed *I*_0_/*I* values around 1 in the presence of six non-specific targets. This clearly indicates that under the tested circumstances, the 6C-miR-21–10bp probe is able to specifically detect its target, miR-21. Taken together, the presented results show that with the chosen system it is possible to balance strong fluorescence, target sensitivity and target specificity, by modulating the length of the fold-back anchor.

### Locking-to-unlocking strategy can be generally applied to design probes for human miRNAs

**Figure 6.**
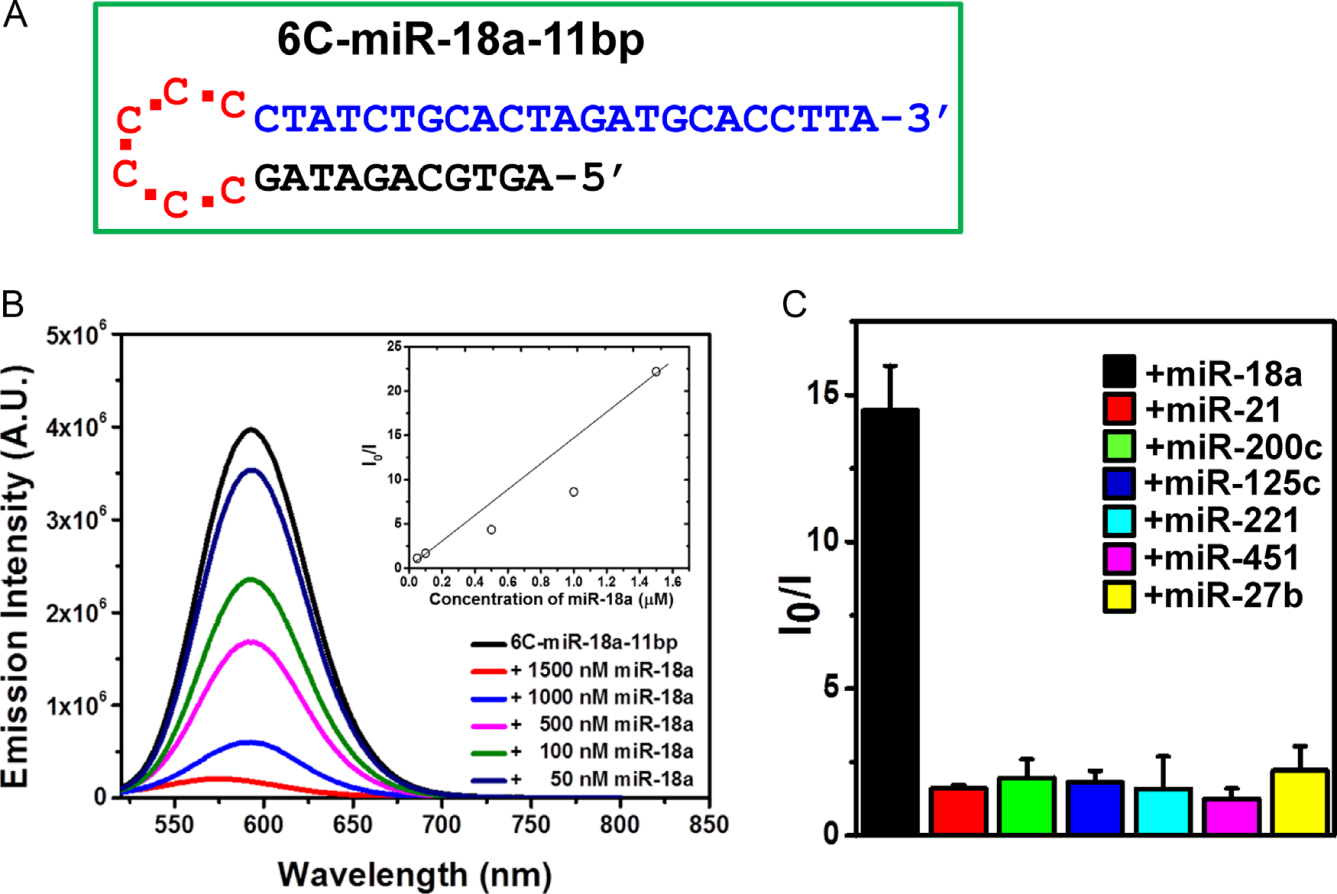
(**A**) Sequence and plausible structure of the 6C-miR-18a-11bp DNA probe. Red sequences: cytosine-loop (6C), blue sequences: target miRNA sensing sequence (miR-18a) and black sequences: the anchor sequence complementary to target sensing sequence. (**B**) Fluorescence intensity of the 6C-miR-18a-11bp DNA probe after addition of target miR-18a in a concentration ranging from 0 to 1.0 μM. The fluorescence spectra were recorded, exciting at 520 nm. The inset shows the Stern–Volmer plot. (**C**) Specificity assessment of the 6C-miR-18a-11bp DNA probe towards different miRNAs measured as the emission spectra obtained following excitation at 520 nm. 1.5 μM of 6C-miR-18a-11bp probe was mixed with 1.5 μM of miR-18a (black bar), miR-21 (red bar), miR-200c (green bar), miR-125c (blue bar), miR-221 (sky blue bar), miR-451 (pink bar) and miR-27b (yellow bar). The data were collected as average value of three individual experiments with ±standard deviation (*n* = 5).

MiR-18a has important roles in controlling gastric cancer growth and angiogenesis (35) and miR-27b is known to regulate numerous types of cancers such as breast and ovarian (36). These miRNAs can thus be useful biomarkers for cancer diagnosis and therefore, functional DNA/AgNCs probes for miR-18a and miR-27b are attractive objectives. Based on the results presented for the fold-back anchored DNA/AgNCs probe for miR-21, we further designed analogous probes for miR-18a and miR-27b. As shown in Figure 6A, in the probe 6C-miR-18–11bp the length of the fold-back anchor was adjusted to give a similar *T*_m_ value to that of 6C-miR-21–10bp. By scanning the excitation spectrum from 340 to 720 nm, we investigated whether the 6C-miR-18–11bp is able to embed highly emissive AgNCs (Supplementary Figure S7). Interestingly, 6C-miR-18–11bp generated very strong orange fluorescence when it was excited at 510 nm. Furthermore, the emission intensity was 2-fold higher than the yellow fluorescence of 6C-miR-21–10bp. The target recognition ability of 6C-miR-18–11bp was tested by adding miR-18a in a concentration dependent manner. As expected, addition of increasing amounts of miR-18a, led to a gradual drop of the orange fluorescence of 6C-miR-18–11bp, revealing a linear dependence of the *I*_0_/*I* intensity versus miR-18a target concentrations (inset shows that the Stern–Volmer plot of the data in Figure 6B). Thus, with 6C-miR-18–11bp is it feasible to detect the presence of target miR-18 at least above 50 nM (Figure 6B). Moreover, we tested the target specificity of the 6C-miR-18–11bp probe against a non-specific miRNA background, by incubating the 6C-miR-18–11bp probe with several non-target miRNAs, miR-200c, miR-125c, miR-221, miR-27b, miR-451 and miR-21 (Figure 6C). Indeed, the 6C-miR-18–11bp probe selectively recognized only its specific target, miR-18a, from a mixture of other miRNAs. As shown in Figure 6C, the 6C-miR-18–11bp probe shows *I*_0_/*I* value of 14 when it encounters its target, implying a high sensitivity of this probe. In a similar manner a probe for miR-27b was designed and named 6C-miR-27b-4bp (Supplementary Figures S8, S9 and corresponding description). These results clearly show that at least the three tested probes with the fold-back anchor design can be highly functional to detect target miRNAs.

### miRNA detection assay

For the miRNA detection assay, we added various concentrations of respective miRNAs to corresponding DNA probes and incubated at 95°C for 10 min followed by incubation for 20 min at 25°C for annealing in the presence of 20 mM Tris-acetate buffer and 25 mM NaNO_3_. For the miRNA specificity assay, 15 μM of different miRNAs were added to 15 μM DNA probes and incubated for 20 min at 25°C. The DNA probes, with or without miRNA, were mixed with AgNO_3_ (250 μM) and NaBH_4_ (250 μM) (1:17:17) to a final volume (50 μl). Before measuring the fluorescence, the volume was increased from 50 to 500 μl for all RNA–miRNAs. We here designate the concentrations of nucleic acids, buffer and salts in the final reaction volume (50 μl).

### Total RNA extraction from cell lines and small RNA northern blot

Total RNA was isolated from HEK-293, HepG2, PANC-1 and MIA PaCa-2, HeLa and MCF-7 cell lines using TRI Reagent Solution (Ambion). RNA concentrations were measured using a NanoDrop ND 1000 spectrophotometer. The small RNA sample (5 μg) of each cell line was mixed with 5 μl of gel loading buffer (Ambion) and resolved on denaturing 15% polyacrylamide gels containing 7.5 M urea. The separated RNA samples were transferred onto a positively charged Amersham Hybond-N+ nylon membrane (GE Healthcare) using a Trans-Blot SD Semi-Dry Electrophoretic Transfer Cell (Bio-Rad). Next, the membranes were further crosslinked using UV irradiation. Before hybridization, the membranes were pre-hybridized for at least 30 min at 40°C in ULTRAhyb®-Oligo hybridization buffer (Ambion). Next, the biotin-labeled probes (complementary to target miRNAs) were added to the hybridization buffer. The membranes were hybridized for 12–16 h at 40°C with gentle shaking and subsequently rinsed with washing buffer (1× SSC, 0.1% SDS) three times for approximately 15 min total at room temperature. The biotin-labeled probes were detected using a Chemiluminescent Nucleic Acid Detection Module Kit (Thermo Scientific, USA). After rinsing, the membranes were blocked again with blocking buffer for 15 min with gentle shaking at room temperature followed by incubation for an additional 15 min with hybridization buffer containing stabilized streptavidin-HRP conjugate. After washing three times, the membranes were equilibrated in substrate equilibration buffer for 5 min. Finally, the membranes were placed in a clean container, covered completely with working solution, and incubated for 5 min in the dark. A working solution was prepared with luminol/enhancer solution and stable peroxide solution. The membranes were placed in a cassette with X-ray films and exposed for different periods of time (depending on the desired signal intensity). For miRNA detection using DNA probes, 5 μg of total RNA (total RNA was redissolved in RNAse free distilled water, instead of a 50% formamide solution, in the final step of purification) was incubated with the respective DNA probes (15 μM). This was followed by the incubation and at 95°C for 10 min. This was followed by the same AgNCs creation procedure as described previously.

### Circular dichroism and absorbance spectroscopy

All CD spectra were recorded twice on a Jasco J-815 spectropolarimeter, in the data interval 320–240 nm with a 1 nm step and a scan rate of 50 nm/min, a 4 s integration time and a 2 nm band width setting. A sample of 180 μl was used in a 1 mm Hellma Quartz cell. Spectra were collected at 25°C. For all spectra equivalent reference spectra were recorded and subsequently subtracted in the Jasco Software Spectra Analysis, where a Savitzky–Golay smoothing was also applied. UV-vis spectra were obtained on a Shimadzu UV-3600 absorbance spectrophotometer using a microvolume 10 mm quartz cell from Hellma Analytics.

### HPLC separation

HPLC separations were performed on a system consisting of two Waters 510 pumps equipped with an automated gradient controller model 680. UV-vis detection was made using a Water 990 photodiode array detector. The separation was performed on a C18 column (120 Å, 3 μm) from FeF Chemicals with the dimensions 4.6 × 150 mm^2^. The separation buffer used was modified from the previous report by Schultz and Gwinn (30). Buffer A consists of 5% methanol, 2.4% trimethylamine acetate (TEAA) in water at pH 7.0 and Buffer B consists of 60% methanol in 2.4% TEAA in water at pH 7.0. All buffers were degassed before use. The sample volume (200 μl) was injected at 100% buffer A and (after 3 min) the separation was performed using a linear gradient from 0% to 85% buffer B over 30 min with a flowrate of 1 ml per minute. Fractions of 1 ml were collected during the run and afterwards the fluorescence was measured on a Jasco FP6300 spectroflurometer at 25°C.

## RESULTS AND DISCUSSION

### Initial studies on hairpin DNA templates

Finding a universal design for miRNA sensing based on DNA/AgNCs, poses an interesting challenge, as the exact interaction between the silver nanoclusters and the nucleic acid is not known in detail. Thus, the optimal folding state of the oligonucleotide for stabilization of a fluorescent species remains to be elucidated. As previously mentioned, there were disadvantages inherent in the design strategy reported by us so far (22,27,31,32). To proceed with an improved design, a strategy using a C/G rich stem with a target sensing loop as reported by Xia *et al*., was interesting, because the hybridization of a target strand could fully disrupt the hairpin structure, leading to an emission drop (33). By applying this approach, we designed and tested two probes—MiR-21–6C/6G and MiR-21-(2CT)^2^/(2GA)^2^—to detect miR-21 (Supplementary Figure S1A and C). In these two cases however, the probes were not capable to generate strong fluorescence. Instead, we proceeded with an alternative design approach: hairpin DNA templates with a perfectly complementary stem (a complementary hairpin) connected by a multi-cytosine loop, which were highly capable of generating strong fluorescence, see Supplementary Figure S2.

Initially, the number of cytosine bases in the loop structure necessary to obtain a strong fluorescence signal in the absence of target miRNA was investigated. As shown in Supplementary Figure S2A, four, six or eight deoxycytidine loops was tethered to a double DNA strand (miR-21/complementary), indicated by 4C-miR-21–22bp, 6C-miR-21–22bp and 8C-miR-21–22bp, respectively. To test the photo-luminescent features of the probes, the emission profiles were recorded following excitation from 300 to 720 nm at every 20 nm, see Supplementary Figure S2B–D. While 4C-miR-21–22bp and 8C-miR-21–22bp, were unable to yield strong fluorescence at any excitation wavelength, 6C-miR-21–22bp showed a strong fluorescence when excited at 480 nm. To determine the most efficient number of cytosines in the loop in other systems, several probes with a 4C, 6C, or 8C loop were tested. The probes with a 4C loop were never able to fluoresce, while the 6C loop consistently was functional in forming fluorescent species in all tested probes. In the case of a longer loop, the 8C tethered probes were irregular in the generation of emissive AgNCs species (see Supplementary Figure S3). With these results, the 6C loop was chosen as the standard in the further design of AgNCs probes.

### Locking-to-unlocking system: fold-back anchored structure notably increases target sensitivity

**Scheme 1.**
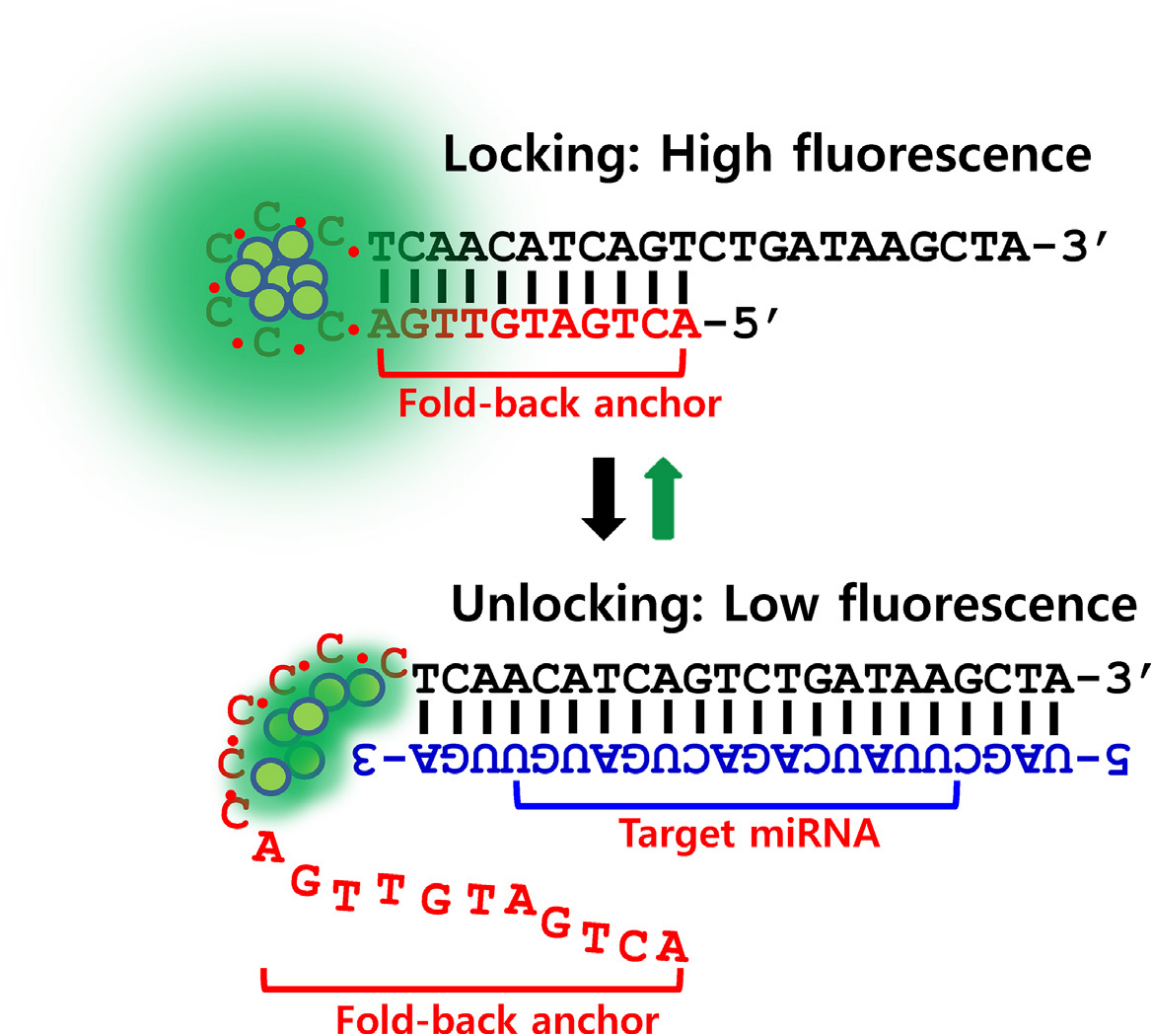
A cartoon of locking-to-unlocking system.

It was further tested whether the 6C-miR-21–22bp probe was competent to detect its target, miR-21. However, irrespective of a range of experimental conditions such as extensive buffer optimizations, the fluorescence of 6C-miR-21–22bp probe was hardly affected by the addition of target miR-21, implying that the target sensibility was insufficient, see Figure 2A. Although many studies have reported DNA/AgNCs probes with hairpin structure that are able to detect the desired target, in our case, the hairpin probe with a perfect complementary 22 nt stem was not optimal. Seemingly, the perfect complementary of the 22 nucleotides of the stem to the target sensing sequence could be too thermodynamically stable to be efficiently disrupted by target hybridization with miRNA. The results are also supported by previous report by the studies on the thermodynamics of DNA/RNA and DNA/DNA duplex (34). In the study, the authors showed that the thermodynamic stability of a DNA/RNA duplex is slightly higher than that of DNA/DNA duplex with same sequences (e.g. *T*_m_ of a DNA/DNA is 34°C, and *T*_m_ of a DNA/RNA with same sequence is 35.5°C in a given salt condition (34)). Based on this evidence, it can confidently be suggested that the miR-21 has to compete with the complementary anchor of 6C-miR-21–22bp, with exactly same sequence as miR-21 only in DNA form. Therefore, without a thermodynamic advantage over the anchor sequences, miR-21 (RNA) can be hardly sensed by 6C-miR-21–22pb. However, by shorting the opposite strand of target sensing strand, the optimal length of hybridization overlap could be obtained, allowing both a strong fluorescence and a high target affinity. As shown in Figure 1A, a series of hairpin probes were prepared by gradually shortening the overlap sequence (we refer to this as the ‘anchor’ hereafter). According to the number of base-paired nucleotides of the anchor, we named the probes 6C-miR-21–11bp, 6C-miR-21–10bp, 6C-miR-21–9bp, 6C-miR-21–8bp, 6C-miR-21–7bp and 6C-miR-21–6bp (Figure 1 and Supplementary Figures S4–S6). Because the thermodynamic stability of a hairpin structure is basically correlated to the strength of base-pairs in its stem, it was expected that the reduction of base-pairs might result in reduction of emission intensity. Indeed, the strong yellow fluorescence of 6C-miR-21–22bp was decreased around 2-fold (3 x10^6^) by shortening of anchor sequence into 11 nucleotides (6C-miR-21–11bp), and likewise in the other shortened 6C-miR-21 probes (Figure 1B). For instance, compared to 6C-miR-21–22bp, the emission intensity was diminished 10-fold in 6C-miR-21–6bp, showing the importance of the anchor in generating or stabilizing emissive AgNCs. Based on this result, we investigated whether the target sensitivity was enhanced in probes with a shortened anchor. In the presence of target miR-21, the emission intensity of 6C-miR-21–22bp was diminished less than 2-fold (max *I_0_*/*I* = 1.66) (Figure 2A). However, 6C-miR-21–11bp and 6C-miR-21–10bp showed values for *I_0_*/*I* of 3.29 and 10.5, respectively (Figure 2B and C). This result indicated that the target sensitivity was increased 2-fold in 6C-miR-21–11bp and 10-fold in 6C-miR-21–10bp as compared to the value of 6C-miR-21–22bp. We further tested the target sensitivity of 6C-miR-21–9bp and 6C-miR-21–8bp and these probes showed values of *I_0_*/*I* of 9.8 and 7.0, respectively (Figure 2D). Although 6C-miR-21–9bp displayed relatively useful target sensitivity, further testing was performed on 6C-miR-21–10bp because of its slightly higher level of fluorescence in the absence of target (See Scheme 1).

Interestingly, the spectral parameters for 6C-miR-27b-4bp are close to those found for the first reported DNA/AgNCs probe, DNA-12nt-RED-160, although the sequence and secondary structure are different (see Supplementary Figure S10) (22). Based on the coincident patterns between 540 and 560 nm, we carefully speculated that 6C-miR-27b-4bp may embed at a species of AgNCs that is highly similar to one of AgNCs in DNA-12nt-RED-160. It is noteworthy, that the three probes designed with the fold-back anchor, yield three different emission colors, while the oligonucleotide structure is expected to be fundamentally similar, with a simple 6C loop and a duplex overlap region. The results indicate not only the presence of at least two different 3D structures of deoxycytidine loops resulting in the differences in excitation/emission spectra (37) but it also shows a subtle role of the specific sequence arrangement and length of the anchor region for the formed AgNCs and their spectral parameters.

### Fold-back anchor probes are functional to detect target miRNAs in total RNA from human cancer cells

**Figure 7.**
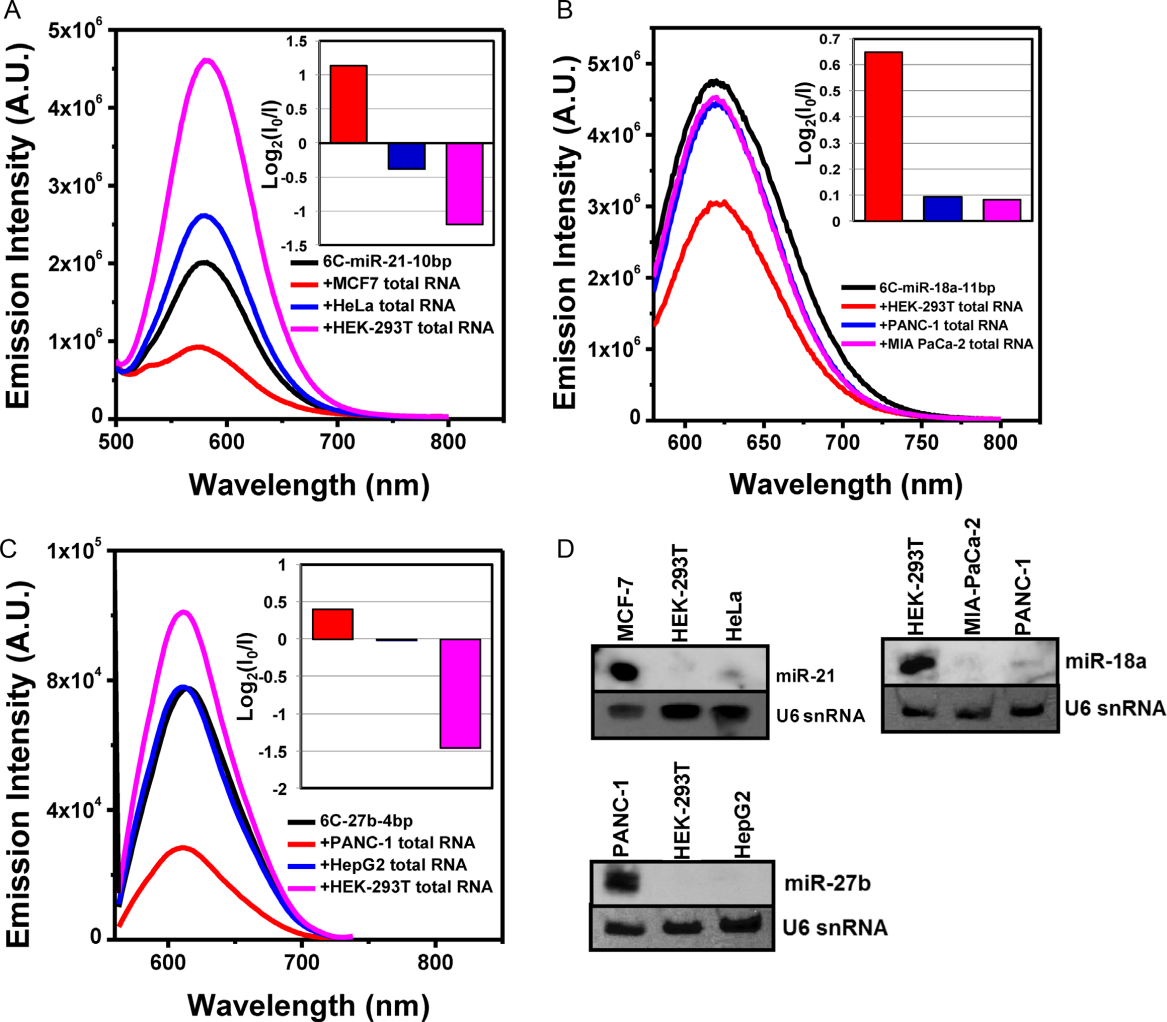
(**A**) Emission spectra (excited at 480 nm) of 6C-miR-21–10bp probe without (black curve) or with 5 μg of total RNA from MCF-7 (red curve), HeLa (blue curve) and HEK-293T (pink curve) cell lines. The Inset shows the log_2_ (*I*_0_/*I*) ratio of each sample. (**B**) Emission spectra (excited at 560 nm) of 6C-miR-18a-11bp probe without (black curve) or with 5 μg of total RNA from HEK-293T (red curve), PANC-1 (blue curve) and MIA PaCa-2 (pink curve) cell lines. The inset shows the log_2_ (*I*_0_/*I*) ratio of each sample. (**C**) Emission spectra (excited at 560 nm) of the 6C-miR-27b-4bp probe without (black curve) or with 5 μg of total RNA from PANC-1 (red curve), HepG2 (blue curve) and HEK-293T (pink curve) cell lines. The inset shows the log_2_ (*I*_0_/*I*) ratio of each sample. (**D**) Small RNA blot analyses show the level of miR-21 (D-1), miR-18a (D-2) and miR-27b (D-3) in the tested cell lines. The level of U6 snRNA was used as a loading control.

To challenge the functionality of the three probes in a complex biological matrix *in vitro*, we investigated the target sensitivity of the probes with total RNA samples extracted from human cancer cell lines. The target genes of miR-21 are involved in the suppression of many cancers such as breast, ovaries, colon, lung, brain, prostate, pancreas and thyroid. Therefore, many studies have evaluated that circulating miR-21 can be a biomarker of many carcinomas, and an excellent candidate early diagnosis. MCF-7 is a breast cancer cell line which has been widely used for cancer research where the expression level of miR-21 is highly up-regulated (38–41). Thus, we tested the level of miR-21 in MCF-7 using 6C-miR-21–10bp probe. HEK-293T is the human embryonic kidney cell line with SV40 Large T-antigen and HeLa is an immortal cell line from cervical cancer. These last two cell lines were used as negative controls. By applying 5 μg of total RNA, we monitored the yellow fluorescence of the 6C-miR-21–10bp probe. As shown in Figure 7A, the emission intensity of the miR-21 probe was notably diminished, when the total RNA from MCF-7 was added (red curve). In contrast, the fluorescence was notably increased by addition of the total RNA from HEK-293T or HeLa cell lines (blue and pink curve), implying no target presence in the samples (Figure 7A). We previously suggested that the incensement of fluorescence in the absence of target as a strong advantage of ‘turn-off’ method (32). To verify the results by another method the level of miR-21 in the tested cell lines was determined using small RNA blot analysis. Indeed, miR-21 was dramatically up-regulated only in the MCF-7 cell line, while the other two control lines showed no expression of miR-21 (Figure 7D-1). These results clearly demonstrates that the fold-back anchor probe for miR-21 successfully distinguished its specific target from thousands of non-specific RNA molecules in a total RNA extraction. Similarly, we tested 6C-miR-18–11bp and 6C-miR-27b-4bp probes with total RNAs from specific cell lines. A recent study (35,42) reported that HEK-293T cell lines maintain a substantial expression level of miR-18a whereas miR-21 and miR-27b are almost untraceable, hence we used total RNA from HEK-293T to validate the 6C-miR-18–11bp probe. In the case of 6C-miR-27–4bp, we examined the functionality of the probe with the PANC-1 cell line, which originates from non-endocrine pancreatic cancer cells (43). Several additional cell lines, MIA PaCa-2 and HepG2 were used as negative controls for each probe, respectively. The former is a type of human pancreas carcinoma and the latter is a perpetual cell line of human liver cancer. As shown Figure 7B, the orange fluorescence of 6C-miR-18–11bp notably decreased when it was mixed with total RNA from HEK-293T cell lines. The orange fluorescence was not dramatically influenced by adding total RNA from PANC-1 or MIA PaCa-2 cell lines (see blue and pink curve of Figure 7B). This result indicated that miR-18a is expressed only in HEK-293T cell lines. Indeed, miR-18a was only detected in HEK-293T cell lines when we cross-validated with northern blot analysis, confirming the accuracy of 6C-miR-18–11bp probe (Figure 7D-2). For similar testing of the 6C-miR-27b-4bp probe, the emission was monitored under the presence of total RNA from PANC-1 cell lines. The red fluorescence of 6C-miR-27b-4bp probe was diminished 2-fold by adding 5 μg of total RNA from PANC-1, and the fluorescence was maintained or increased when 6C-miR-27b-4bp was mixed with total RNA from two control cell lines, HepG2 and HEK-293T, respectively (Figure 7C). This result confirms that the expression level of miR-27b is higher in PANC-1 than in the other two controls. As shown in Figure 7D-3, miR-27b is detected only in PANC-1 cell lines, validating the functionality of when 6C-miR-27b-4bp probe.

Taken all together, we have shown the functionality and practicality of the presented fold-back anchor probes for detection of their specific targets. The readouts of all three tested probes, which tell the presence of each target miRNA with a reliable accuracy, were in every case consistent with the results of obtained from northern blot analysis, clearly suggesting the usability of this novel strategy, as an easier alternative approach.

## CONCLUSION AND PERSPECTIVES

We have demonstrated that the DNA/AgNCs-based miRNA detection can be a complementary measure to many conventional methods for miRNA detection. This novel method has several advantages; for instance, the fluorescence of silver nanoclusters is much safer than the radioactivity of isotope labeling and requires fewer steps in using than that of Digoxigenin (DIG) labeling. Compared to the highly sensitive method, real-time polymerase chain reaction method, our method is limited in detecting target in the range of conventional RNA blot analysis. However, the presented method relies on the novel phenomenon silver nanocluster fluorscence, which may be developed further in the coming years. Previously, the importance of secondary structures in embedding emissive AgNCs was first suggested by showing the functionality of hairpin structured DNA templates. The hairpin structured DNA templates harbor deoxycytidines in the loop region where emissive AgNCs could be clustered (27,44). Similarly, we found that the formation of highly emissive AgNCs was not only determined by a given scaffold sequence but also influenced by the secondary structure—mismatch self-dimer or hairpin—of the particular probe (27). We further demonstrated that the emission drop of DNA/AgNCs probe is caused by the structural disruption of a DNA/AgNCs probe upon target hybridization (22,27). This mismatch structure strategy represents a useful approach for miRNA detection in selected cases (22,44). However, in a series of DNA/AgNCs probes designed for various miRNAs, we found that the approach using the mismatched hairpin and self-dimer structures is not always able to obtain functional DNA/AgNCs. For instance, 5 of 40 designed DNA/AgNCs probes were neither successful in generating strong emission nor in sensing target, and the rate of failure (12.5%) was unfavorable for practical applications. To overcome these drawbacks in the design, DNA/RNA chimera nucleic acids were adopted as templates, by which fluorescence and target accessibility of several probes could be dramatically improved (31). We interpreted that the freedom of rotation in sugar backbone of RNA is an inbuilt advantage of DNA/RNA chimera template for embedding emissive AgNCs species. However, in following studies, this strategy also disclosed several drawbacks; such as unpredictable and non-standardized structure formation. To build a high throughput miRNA detection system, neither of the strategies is ideal for a general procedure, uniformly applicable regardless of the exact target sequence.

To confine the secondary structure of DNA templates to a single unitary structure, we chose the hairpin structure with a deoxycytidine loop. However, as shown in Figure 2, a hairpin structure with a high thermo-stability (perfect complementary stem) strongly hinders the target accessibility to the probe; although it may show a strong fluorescence, this read-out is not affected by miRNA binding. However, by introducing a shorter fold-back anchor, we managed to reduce the thermal stability of hairpin structure yet retaining strong fluorescence and simultaneously enabling the probe to recognize its miRNA target. According to the nearest-neighbor parameters, dCG (dGC), dGC (dCG) and dGG (dCC) pairs has the lowest free energy Δ*G*^37^ = -2.8, -2.3 and -2.1 kJ/mole, respectively (45). Based on the studies, we found that the length of anchor has to be adjusted by the free energy prediction. In contrast to the previous design strategies—mismatch self-dimer or hairpin (27) and DNA/RNA chimera (32)—, this locking-to-unlocking system allows us to efficiently predict the secondary structures of probes and therefore it could alleviate the demanding functional analysis by trial and error, in the design of novel probes. Based on this study, we envision developing a computerized design program that suggests an optimized fold-back anchor sequence for each specific miRNA. Ultimately, the aim would be to produce a full set of DNA/AgNCs probes covering most of miRNAs in human and plants (44).

## Supplementary Material

SUPPLEMENTARY DATA
