# Supplementary material for: Locking-to-unlocking system is an efficient strategy to design DNA/silver nanoclusters (AgNCs) probe for human miRNAs
Source: Nucleic Acids Res. 2015 Dec 17;44(6):e57. doi: 10.1093/nar/gkv1377 (PMC4824086; doi:10.1093/nar/gkv1377)
Supplement: SUPPLEMENTARY DATA [file supp_44_6_e57__index.html]

Locking-to-unlocking system is an efficient strategy to design DNA/silver nanoclusters (AgNCs) probe for human miRNAs — SUPPLEMENTARY DATA 

# Locking-to-unlocking system is an efficient strategy to design DNA/silver nanoclusters (AgNCs) probe for human miRNAs

## SUPPLEMENTARY DATA

- SUPPLEMENTARY DATA
