# Supplementary material for: Locking-to-unlocking system is an efficient strategy to design DNA/silver nanoclusters (AgNCs) probe for human miRNAs
Source: Nucleic Acids Res. 2015 Dec 17;44(6):e57. doi: 10.1093/nar/gkv1377 (PMC4824086; doi:10.1093/nar/gkv1377)
Supplement: SUPPLEMENTARY DATA [file supp_gkv1377_nar-01573-met-g-2015-File002.docx]

**Supplementary Information**

**Locking-to-unlocking system is an efficient strategy to design DNA/silver nanoclusters (AgNCs) probe for human miRNAs**

^1^Pratik Shah†, ^3^Suk Won Choi†, ^3^Ho-jin Kim†, ^1^Seok Keun Cho, ^3^Yong-Joo Bhang, ^3^Jong Cheol Ahn, ^2^Peter [Waaben Thulstrup](http://pubs.acs.org/action/doSearch?action=search&author=Thulstrup%2C+P+W&qsSearchArea=author)*, ^2^Morten Jannik Bjerrum*, and ^1, 4^Seong Wook Yang*

1UNIK Center for Synthetic Biology / Plant Biochemistry Laboratory, Department of Plant and Environmental Sciences, University of Copenhagen, Thorvaldsensvej 40, DK-1871 Frederiksberg C, Copenhagen, Denmark

^2^Department of Chemistry, University of Copenhagen, Universitetsparken 5, DK-2100, Copenhagen, Denmark

^3^Seoulin bioscience Co. Ltd. 4F. #A, KOREA BIO PARK, 700, Daewangpangyo-ro, Bundang-gu, Seongnam-si, Gyeonggi-do, Korea

^4^Department of Systems Biology, College of Life Science and Biotechnology, Yonsei University,

*Correspondence to [swyang@plen.ku.dk](mailto:swyang@plen.ku.dk), [pwt@chem.ku.dk](mailto:pwt@chem.ku.dk), and mbj@chem.ku.dk

**SUPPLEMENTARY DESCRIPTION**

In a similar manner a probe for miR-27b was designed and named 6C-miR-27b-4bp (Supplementary Figure S8A). As for the other probes the excitation spectrum was scanned from 340 nm to 720 nm, and the highest emission wavelength of 6C-miR-27b-4bp was identified (Supplementary Figure S9). In the case of 6C-miR-27b-4bp, we observed a common red fluorescence when excited at 540 nm and the intensity reaches around 1.5 x10^6^ (Supplementary Figure S8B). As the logical next step, the functionality of 6C-miR-27b-4bp was verified by the observation of a gradual drop of the red fluorescence of 6C-miR-27b-4bp in response to the increasing concentrations of miR-27b, that follows a linear dependence of the *I_0_/I* ratio versus miR-27b target concentration (Inset shows that the Stern-Volmer plot of the data in Supplementary Figure S8B). The target specificity of the 6C-miR-27b-4bp probe against non-specific miRNA backgrounds was also verified, and it was shown similarly to the other probes, the 6C-miR-27b-4bp probe effectively recognized only its specific target miR-27b from non-specific targets, displaying an *I*_0_*/I* value of 8 when it encounters its target (Supplementary Figure S8C).

**\**

SUPPLEMENTARY FIGURE S1. A) Sequence and plausible structure of the miR-21-5C/5G DNA probe. The target sensing sequences (black) was reconstituted with extra stem sequences (red) to induce the formation of hairpin structure. B) Emission spectra of miR-21-6C/6G (1.5 µM) probe. C) Sequence and plausible structure of the miR-21-(2CT)2/(2GA)2 DNA probe. The target sensing sequences (black) was reconstituted with extra stem sequences (red) to induce the formation of hairpin structure. D) Emission spectra of miR-21-(2CT)2/(2GA)2 (1.5 µM) probe. The spectra were recorded by exciting from 300-720 nm in 20 nm steps. The signals between 300 nm and 500 nm correspond to Raman scattering from water.

SUPPLEMENTARY FIGURE S2. Effect of cytosine number in a loop for embedding emissive AgNCs. A) Sequence and plausible structure of DNA/AgNCs probes for miR-21 with a different loop size. Each DNA probe sequence is given in three colors. Red squences: cytosine-loop (nC), blue sequences: target miRNA sensing sequence (miR-21), and black sequences: anchor sequence (22bp), complementary to target sensing sequence. B) Emission spectra of 4C-miR-21-22bp (1.5 µM) probe (4 cytosines in a loop). C) Emission spectra of 6C-miR-21-22bp (1.5 µM) probe (6 cytosines in a loop). D) Emission spectra of 8C-miR-21-22bp (1.5 µM) probe (8 cytosines in a loop). The spectral heterogeneity / change in emission maximum with high excitation wavelengths indicate the presence of alternate types of AgNCs. The signals between 300 nm and 500 nm correspond to Raman scattering from water.

SUPPLEMENTARY FIGURE S3. Effect of cytosine number in a loop and unstable stem on the generation of emissive AgNCs. A) Sequence and plausible structure of DNA/AgNCs probes for miR-21 with a different anchor length. Each DNA probe sequence is given in three colors. Red squences: cytosine-loop (nC), blue sequences: target miRNA sensing sequence (miR-21), and black sequences: short anchor sequence (11bp). B) Emission spectra of 4C-miR-21-11bp (1.5 µM) probe (4 cytosines in a loop). C) Emission spectra of 6C-miR-21-11bp (1.5 µM) probe (6 cytosines in a loop). D) Emission spectra of 8C-miR-21-11bp (1.5 µM) probe (8 cytosines in a loop). The spectral heterogeneity / change in emission maximum with high excitation wavelengths indicate the presence of alternate types of AgNCs. The signals between 300 nm and 500 nm correspond to Raman scattering from water.

SUPPLEMENTARY FIGURE S4: Effect of anchor length on the generation of emissive AgNCs. A) Emission spectra of 6C-miR-21-11bp (1.5 µM). B) Emission spectra of 6C-miR-21-10bp (1.5 µM). The spectra were recorded by exciting from 300-720 nm in 20 nm steps. The spectral heterogeneity / change in emission maximum with high excitation wavelengths indicate the presence of alternate types of AgNCs. The signals between 300 nm and 500 nm correspond to Raman scattering from water.

SUPPLEMENTARY FIGURE S5: Effect of anchor length on the generation of emissive AgNCs. A) Emission spectra of 6C-miR-21-9bp (1.5 µM). B) Emission spectra of 6C-miR-21-8bp (1.5 µM). The spectra were recorded by exciting from 300-720 nm in 20 nm steps. The spectral heterogeneity / change in emission maximum with high excitation wavelengths indicate the presence of alternate types of AgNCs. The signals between 300 nm and 500 nm correspond to Raman scattering from water.

SUPPLEMENTARY FIGURE S6: Effect of anchor length on the generation of emissive AgNCs. A) Emission spectra of 6C-miR-21-7bp (1.5 µM). B) Emission spectra of 6C-miR-21-6bp (1.5 µM). The spectra were recorded by exciting from 300-720 nm in 20 nm steps. The signals between 300 nm and 500 nm correspond to Raman scattering from water.

SUPPLEMENTARY FIGURE S7: Emission spectra of 6C-miR-18a-11bp (1.5 µM). The spectra were recorded by exciting from 300-720 nm in 20 nm steps. The spectral heterogeneity / change in emission maximum with high excitation wavelengths indicate the presence of alternate types of AgNCs. The signals between 300 nm and 500 nm correspond to Raman scattering from water.

SUPPLEMENTARY FIGURE FIGURE S8. A) Sequence and plausible structure of the 6C-miR-27b-4bp DNA probe. Red sequences: cytosine-loop (6C), blue sequences: target miRNA sensing sequence (miR-27b) and black sequences: the anchor sequence complementary to target sensing sequence. B) Fluorescence intensity of the 6C-miR-27b-4bp DNA probe after addition of target miR-18a in a concentration ranging from 0 to 1.0 µM. The fluorescence spectra were recorded, exciting at 560 nm. The inset shows the Stern-Volmer plot. C) Specificity assessment of the 6C-miR-27b-4bp DNA probe towards different miRNAs measured as the emission spectra obtained following excitation at 560 nm. 1.5 µM of 6C-miR-27b-4bp probe was mixed with 1.5 µM of miR-27b (black bar), miR-21 (red bar), miR-200c (green bar), miR-125c (blue bar), miR-221 (sky blue bar), miR-451 (pink bar) and miR-18a (yellow bar). The data were collected as average value of three individual experiments with ±standard deviation (n=5).

SUPPLEMENTARY FIGURE S9: Emission spectra of 6C-miR-27b-4bp (1.5 µM). The spectra were recorded by exciting from 300-720 nm in 20 nm steps. The spectral heterogeneity / change in emission maximum with high excitation wavelengths indicate the presence of alternate types of AgNCs. The signals between 300 nm and 500 nm correspond to Raman scattering from water.

SUPPLEMENTARY FIGURE S10: Comparison of the highest emission spectra of 6C-miR-27b-4bp (1.5 µM) and DNA-12nt-RED-160 (1.5 µM), excited at 540 nm or 560 nm (22).


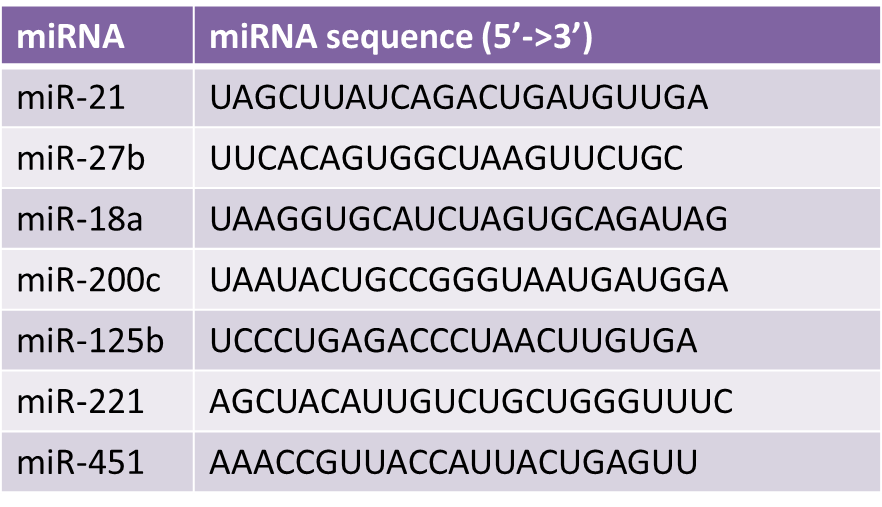


SUPPLEMENTARY TABLE1: Sequence information of target miRNAs and controls
